# Supplementary material for: Toll-Like Receptors-2 and -4 in Graves’ Disease—Key Players or Bystanders?
Source: Int J Mol Sci. 2019 Sep 24;20(19):4732. doi: 10.3390/ijms20194732 (PMC6801632; doi:10.3390/ijms20194732)
Supplement: Supplementary file 1 [file ijms-20-04732-s001.pdf]

Table S1. TLR-2 expression on T and B lymphocytes in the peripheral blood.

Variable	Mean	Median	Minimum	Maximum	Lower quartile	Upper quartile	SD
Percentage values of T CD4+/TLR-2+ (among CD4+ cells) in patients with GD before treatment [%]	1.32	1.31	0.43	3.82	0.93	1.52	0.59
Percentage values of T CD4+/TLR-2+ (among CD4+ cells) in patients with GD after treatment [%]	0.59	0.59	0.43	0.73	0.52	0.65	0.09
Percentage values of T CD4+/TLR-2+ (among CD4+ cells) in the control group [%]	0.26	0.26	0.09	0.42	0.14	0.36	0.11
TLR-2 expression on T CD4+ lymphocytes [MFI] in patients with GD before treatment	227.62	55.25	9.74	1674.35	16.82	189.00	436.27
TLR-2 expression on T CD4+ lymphocytes [MFI] in patients with GD after receiving euthyreosis	172.90	58.40	9.71	1101.04	13.22	182.90	250.75
TLR-2 expression on T CD4+ lymphocytes [MFI] in the control group	129.33	19.41	10.47	631.30	12.00	57.00	224.07
Percentage values of T CD8+/TLR-2+ (among CD8+ cells) in patients with GD before treatment [%]	4.01	4.19	0.43	8.17	1.26	5.48	2.37
Percentage values of T CD8+/TLR-2+ (among CD8+ cells) in patients with GD after treatment [%]	2.35	2.61	0.21	4.51	1.15	3.23	1.27
Percentage values of T CD8+/TLR-2+ (among CD8+ cells) in the control group [%]	1.30	0.71	0.11	4.43	0.31	1.67	1.46
TLR-2 expression on T CD8+ lymphocytes [MFI] in patients with GD before treatment	54.54	25.38	4.87	198.00	23.30	57.33	52.25
TLR-2 expression on T CD8+ lymphocytes [MFI] in patients with GD after receiving euthyreosis	44.90	40.60	6.63	132.70	18.48	57.28	35.62

TLR-2 expression on T CD8+ lymphocytes [MFI] in the control group	33.37	16.11	2.84	131.46	11.83	29.43	43.23
Percentage values of B CD19+/TLR-2+ (among CD19+ cells) in patients with GD before treatment [%]	1.54	1.09	0.31	3.98	0.83	2.11	1.04
Percentage values of B CD19+/TLR-2+ (among CD19+ cells) in patients with GD after treatment [%]	1.32	1.08	0.09	6.41	0.52	1.58	1.33
Percentage values of B CD19+/TLR-2+ (among CD19+ cells) in the control group [%]	0.88	0.62	0.12	3.30	0.36	1.02	0.86
TLR-2 expression on B CD19+ lymphocytes [MFI] in patients with GD before treatment	60.14	56.21	12.52	104.88	38.15	77.12	24.39
TLR-2 expression on B CD19+ lymphocytes [MFI] in patients with GD after receiving euthyreosis	52.03	40.77	11.82	181.58	29.79	62.41	38.77
TLR-2 expression on B CD19+ lymphocytes [MFI] in the control group	50.81	48.47	31.34	81.97	39.97	60.06	13.43

GD Graves' disease, MFI mean fluorescence intensity

Table S2. TLR-4 expression on T and B lymphocytes in the peripheral blood.

Variable	Mean	Median	Minimum	Maximum	Lower quartile	Upper quartile	SD
Percentage values of T CD4+/TLR-4+ (among CD4+ cells) in patients with GD before treatment [%]	0.63	0.62	0.21	0.99	0.43	0.83	0.23
Percentage values of T CD4+/TLR-4+ (among CD4+ cells) in patients with GD after treatment [%]	0.57	0.60	0.05	0.99	0.44	0.73	0.23
Percentage values of T CD4+/TLR-4+ (among CD4+ cells) in the control group [%]	0.24	0.13	0.00	0.82	0.08	0.33	0.25
TLR-4 expression on T CD4+ lymphocytes [MFI] in patients with GD before treatment	430.74	432.00	8.99	954.00	138.86	677.20	294.78
TLR-4 expression on T CD4+ lymphocytes [MFI] in patients with GD after receiving euthyreosis	412.33	312.66	7.29	939.29	91.96	715.65	355.46
TLR-4 expression on T CD4+ lymphocytes [MFI] in the control group	267.74	100.00	11.74	752.00	52.71	466.35	263.30
Percentage values of T CD8+/TLR-4+ (among CD8+ cells) in patients with GD before treatment [%]	2.74	3.06	0.62	4.35	1.69	3.43	1.07
Percentage values of T CD8+/TLR-4+ (among CD8+ cells) in patients with GD after the treatment [%]	2.55	2.48	0.08	5.01	0.88	3.70	1.50
Percentage values of T CD8+/TLR-4+ (among CD8+ cells) in the control group [%]	0.98	0.49	0.02	4.35	0.27	1.09	1.24
TLR-4 expression on T CD8+ lymphocytes [MFI] in patients with GD before treatment	158.92	114.50	8.60	540.09	30.97	215.76	145.89
TLR-4 expression on T CD8+ lymphocytes [MFI] in patients with GD after receiving euthyreosis	133.96	138.50	9.14	275.87	103.09	169.50	54.35

TLR-4 expression ofnT CD8+ lymphocytes [MFI] in the control group	73.38	30.75	10.20	498.74	18.84	89.90	109.67
Percentage values of B CD19+/TLR-4+ (among CD19+ cells) in patients with GD before treatment [%]	1.68	0.96	0.09	7.23	0.45	2.16	1.84
Percentage values of B CD19+/TLR-4+ (among CD19+ cells) in patients with GD after treatment [%]	1.32	1.28	0.11	7.48	0.41	1.81	1.39
Percentage values of B CD19+/TLR-4+ (among CD19+ cells) in the control group [%]	0.69	0.41	0.09	3.28	0.28	0.74	0.78
TLR-4 expression on B CD19+ lymphocytes [MFI] in patients with GD before treatment	88.73	59.77	20.25	284.67	37.98	126.42	71.98
TLR-4 expression on B CD19+ lymphocytes [MFI] in patients with GD after receiving euthyreosis	81.70	57.45	10.62	303.69	37.21	109.49	65.58
TLR-4 expression on B CD19+ lymphocytes [MFI] in the control group	80.70	77.13	42.86	146.60	56.73	100.55	29.74
